# A Single Session of a Digital Health Tool-Delivered Exercise Intervention May Provide Immediate Relief from Pelvic Pain in Women with Endometriosis: A Pilot Randomized Controlled Study

**DOI:** 10.3390/ijerph20031665

**Published:** 2023-01-17

**Authors:** Muhammad Lutfi, Lance C. Dalleck, Claire Drummond, Murray Drummond, Liana Paparella, Caitlin E. Keith, Michael Kirton, Laura Falconer, Lemlem Gebremichael, Caroline Phelan, Christine Barry, Kiara Roscio, Belinda Lange, Joyce S. Ramos

**Affiliations:** 1 Caring Futures Institute, SHAPE Research Centre, Clinical Exercise Physiology, College of Nursing and Health Sciences, Flinders University, Adelaide, SA 5042, Australia; 2 Recreation, Exercise, and Sports Science Department, Western Colorado University, Gunnison, CO 81231, USA; 3 College of Medicine and Public Health, Flinders University, Adelaide, SA 5042, Australia

**Keywords:** virtual reality, telehealth, women’s health, endometriosis, pelvic pain

## Abstract

Background: Endometriosis is a debilitating chronic condition that is commonly associated with chronic pelvic pain, affecting approximately 10% of women of reproductive age worldwide. The general principle of pain management in this population involves both pharmacological and surgical interventions. There is also increasing interest in the use of exercise as an alternative non-pharmacological analgesic, but adherence and accessibility to face-to-face exercise-delivery modalities are poor. This study aims to determine the immediate impact of a single session of ‘supervised’ telehealth-delivered exercise compared to ‘self-managed’ virtual reality (VR)-delivered exercise on pelvic pain associated with endometriosis. Methods: Twenty-two women experiencing pelvic pain due to endometriosis were included and randomized into three groups: (i) VR-delivered exercise group (n = 8); (ii) telehealth-delivered exercise group (n = 8); and (iii) control group (n = 6). The visual analogue scale (VAS) was used to assess the severity of pelvic pain. Results: There was no statistically significant between-group difference (*p* = 0.45) in the participants’ pain score following a single session of the study interventions (VR or telehealth) or the control. However, a ‘medium-to-large’ group x time interaction effect (η^2^ = 0.10) was detected, indicating a more favorable pain score change following a single session of telehealth- (pre-post ∆: +10 ± 12 mm) and VR-delivered exercise (pre-post ∆: +9 ± 24 mm) compared to the control group (pre-post ∆: +16 ± 12 mm). Conclusions: Our study suggests that a single bout of a ‘self-managed’ VR-delivered exercise may be as efficacious as a single session of ‘supervised’ telehealth-delivered exercise in providing immediate relief from pelvic pain associated with endometriosis.

## 1. Introduction

Endometriosis is an estrogen-dependent chronic inflammatory disease that denotes the growth of abnormal endometrial-like tissue outside of the uterine cavity [1]. Globally, it affects approximately 10% of reproductive-age women [2]. Individuals with this condition often present with non-specific symptoms, including pelvic pain [1]. It has serious implications for public health with an estimated economic cost of AUD 7.4 billion in Australia from 2017 to 2018 [3]. The burden of disease and work productivity loss [4] accounts for the majority of this cost [3].

Endometriosis is considered a chronic disease that requires a lifelong management plan [5]. In particular, the current principle of endometriosis treatment lies in pain management [6], including medical treatment, surgery, or a combination of both. In recent years, there has been an increased interest in the use of complementary treatment options when conventional therapies are unsatisfactory [7]. Exercise has been shown to be effective in relieving acute global pain [8,9,10]. However, the adherence and accessibility of face-to-face delivery modalities in general are low. It has also been reported that the COVID-19 pandemic has reduced access to medical care by 47.2% in women with endometriosis, which has caused the worsening of symptoms, such as pelvic pain, anxiety, and depression [11].

There is a growing interest in exploring different exercise-delivery modalities in the treatment of acute pain. Specifically, the use of telehealth has been demonstrated to be as effective in delivering exercise interventions as face-to-face modalities in improving pain, physical activity (PA) levels, activities of daily living, and quality of life [12]. However, to the best of our knowledge, no studies have explored the impact of telehealth-delivered exercise on pelvic pain experienced by women with endometriosis. It has also recently been suggested that telehealth may be a suitable alternative for women with endometriosis experiencing pain that limits their ability to travel to a healthcare provider [13]. Virtual reality (VR) has also been successfully employed for the self-management of various pain conditions [14], specifically in the management of pelvic pain associated with endometriosis [15]. It has been postulated that the use of VR may improve adherence to treatment due to its game-like nature, which creates a motivational environment that encourages patients to actively engage in treatment [16].

While the long-term pain-relieving effects of these digital health tools on global pain have been documented extensively [14,17], there are no studies available that compare the immediate analgesic impact of a ‘supervised’ telehealth-delivered versus a ‘self-managed’ VR-delivered exercise session. The availability of an efficacious self-managed digital health tool is particularly important among those with busy lifestyles or those who live in rural and remote areas with limited access to synchronous healthcare. As such, the role of these digital health tools needs to be explored, with the aim of investigating their potential in providing immediate symptomatic pain relief that is non-pharmaceutical and readily accessible to women experiencing pelvic pain associated with endometriosis.

The aim of this study was to investigate the immediate impact of a single session of supervised telehealth-delivered exercise versus self-managed VR-delivered exercise on pelvic pain in women with endometriosis. We hypothesized that a single session of a self-managed VR-delivered exercise intervention would be as efficacious as the telehealth-delivered exercise bout in reducing pelvic pain in women with endometriosis.

## 2. Materials and Methods

**Figure 1:**
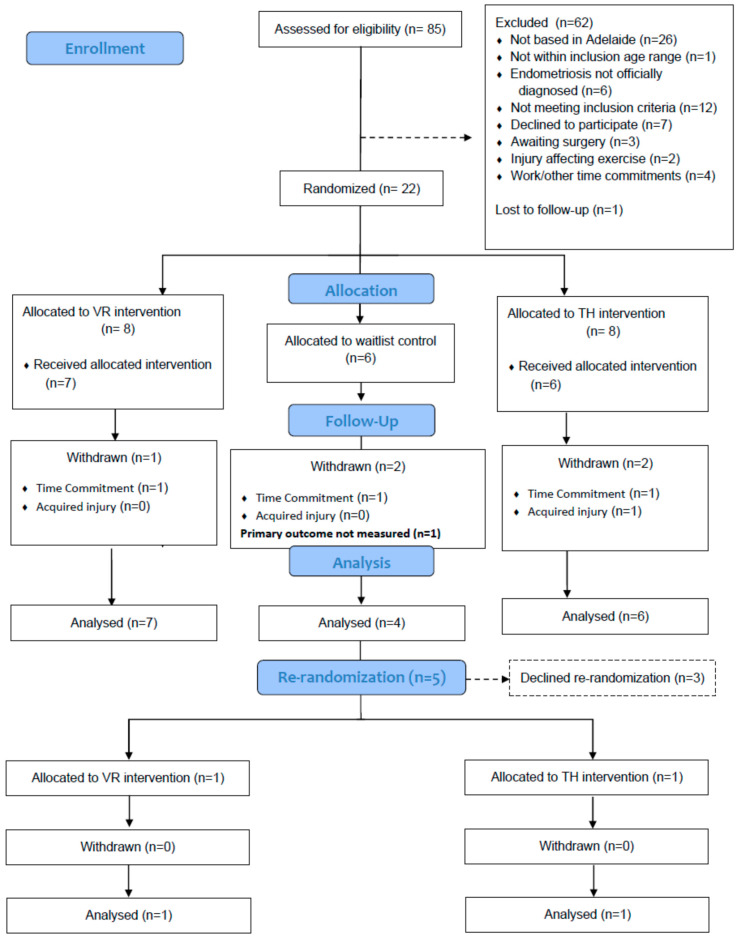
Participant flow chart.

Participants were recruited via advertisements in local general practices, university newsletters, study websites, and other social media. Participants who expressed interest were contacted via a letter of invitation, which explained the purpose of the study. Screening for study eligibility was initiated via an online questionnaire located within the study website and confirmed via a follow-up phone call from a member of the research team. Eighty-five individuals (18–45 years) diagnosed with endometriosis undertook screening (January 2021 to September 2022) for the study eligibility criteria. Figure 1 presents a consort diagram of participant flow throughout the study. Participants with uncontrolled asthma, unstable respiratory, or cardiac conditions; diagnosed with epilepsy or recent history of seizures; limited arm and hand movement that would make it difficult to interact with virtual reality technology; and those with visual impairments (blindness, partial blindness, and visual perceptual problems) were excluded from the study. Twenty-two eligible participants were randomized (stratified by age) to one of the following groups: (i) telehealth-delivered exercise (n = 8); (ii) VR-delivered exercise (n = 8); or (iii) control (n = 6). The randomization procedure was conducted using randomization software employing random permuted blocks. Participants provided written consent to participate in the study. The participants in the intervention groups were required to attend one supervised exercise training session whilst the control group were instructed to continue with their activities of daily living. This study was approved by the Flinders University Human Ethics Research Committee (approval number: 2541).

### 2.1. Acute Pelvic Pain

Acute pelvic pain was evaluated using the 100 mm visual analogue scale (VAS) [18] at baseline and 48 h following a single exercise bout delivered via telehealth or VR, or no intervention (control). The participants were instructed to assume ‘0 mm’ as no pain experienced, whereas 100 mm represented the most pain experienced. The participants were then instructed to mark somewhere on the VAS scale with a perpendicular line to indicate their pain score, after which the distance of the line from 0 mm was measured.

### 2.2. Cardiorespiratory Fitness

Cardiorespiratory fitness was assessed via indirect calorimetry (Metamax, Cortex, Leipzig, Germany) during an individualized graded maximal-exercise test using a cycle ergometer (Ergoline, Germany). The aim of this procedure was to determine the cardiorespiratory exercise intensity at which the telehealth session was delivered. Ventilation, oxygen, and carbon dioxide concentrations were measured during the test via a metabolic cart (MetaMax, Cortex, Leipzig, Germany). A 3 min warm-up was initiated to familiarize the participants to the test protocol (unloaded cycling at 50 to 60 revolutions per minute [RPM]). The maximal-exercise test was initiated using a workload that was harder than the warm-up stage (load individualized at 25–50 W and at 50–60 RPM). Thereafter, the workload was progressively increased every minute by 25 W until the participant could no longer continue. The test was approximately 10–15 min in duration. Blood pressure, heart rate (HR), and rate of perceived exertion (RPE) were monitored throughout the test to ensure the safety of participants.

### 2.3. Telehealth Session

Telehealth-delivered exercise group: Participants completed a 1 h supervised session. HR, RPE, and exercise volume (intensity and duration) during the session were monitored and recorded. HR and RPE were monitored via an HR monitor (Polar Electro, Kempele, Finland) and the Borg 6–20 RPE scale [19], respectively. The exercises included cardiorespiratory exercise and stretching and specific stabilizing exercise (SSE) of muscles within the lumbopelvic area. The cardiorespiratory fitness training used an interval training protocol which consisted of 4 min exercise bouts, repeated three times and separated by two minutes of active recovery. The interval training was preceded by a 3 min warm-up, with a total exercise time of 19 min. The cardiorespiratory fitness intensity was prescribed according to the ventilatory thresholds (VT1 and VT2) determined from the gas parameters acquired from the individualized graded maximal-exercise test. A target HR coinciding with the prescribed ventilatory threshold zone was used to establish a specific exercise training intensity for the telehealth session. The target HR for the single telehealth session was established as follows: target HR = HR range of 10–15 beats per minute (bpm), just below VT1.

### 2.4. Virtual Reality Session

VR-delivered exercise group: Participants completed a 1 h unsupervised session. HR, RPE, and exercise volume (intensity and duration) during the session were monitored and recorded. HR and RPE were monitored via an HR monitor (Polar Electro, Kempele, Finland) and the Borg 6–20 RPE scale [19], respectively. The session consisted of the following: (i) a 10 min VR pain-distraction experience via a list of applications previously shown to reduce pain [20]; and (ii) 50 min of exercise using one of the following applications depending on the participants’ preference and goals (Dance Central, Beat Saber, The Thrill of the Fight, Space Pirate Trainer, Fruit Ninja, OhShape, Racket NX, Table Tennis VR, Racket Fury, Swords of Gargantua, BoxVR, Superhot VR, VZ Fit Play, and VZFit Explorer).

### 2.5. Statistical Analysis

The SPSS version 28 software package (IBM, New York, NY, USA) was used to analyze all data. To determine the suitability of parametric tests, the assumption of normality was tested via the Shapiro–Wilk test. Between-group differences in VAS score changes from pre- to post-study exposures (interventions (VR or telehealth) or control) were determined via repeated-measures analysis of covariance (ANCOVA), with the study exposures assigned as the between-group variables. The baseline VAS scores were entered as the co-variate, and the VAS change scores from pre- to post-acute training session were entered as the dependent variable. The significance level was set at *p* < 0.05. Eta-squared (η^2^) group × time interaction effect sizes were calculated as the between-group sum of squares divided by the total sum of squares and interpreted as follows: ‘small’ effect (<0.01); ‘small-to-medium’ effect (0.01 to <0.10); ‘medium-to-large’ (0.10 to 0.25) [21].

## 3. Results

Nineteen participants (telehealth, n = 7; VR, n = 8; control, n = 4) completed the assessment for the primary outcome of the study (Figure 1). One participant in the VR group was excluded from analysis due to not attending VAS measurements after their acute training bout. No adverse outcomes related to the study were encountered. Table 1 presents the baseline characteristics of the participants within each study group.

### Pelvic Pain

**Figure 2:**
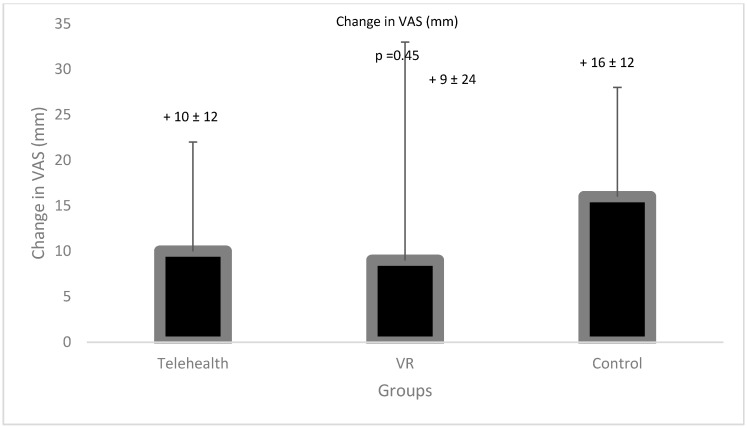
Changes in VAS scores following different types of interventions in women with mild-to-moderate endometriosis.

There were no significant differences in VAS score changes between groups following the acute training interventions (Figure 2, *p* = 0.45). Compared to the control group (+16 ± 12 mm), both telehealth- (+10 ± 12 mm) and VR-delivered exercise (+9 ± 24 mm) interventions showed a lower magnitude of pain score increase from baseline. There was also a ‘medium-to-large’ group × time interaction effect (η^2^ = 0.10) on the VAS scores, indicating lower pelvic pain experienced by women diagnosed with endometriosis following single VR-delivered and telehealth-delivered exercise interventions compared to the control.

## 4. Discussion

This study is the first randomized controlled study to compare the impacts of a single bout of telehealth- and VR-delivered exercise session on pelvic pain in women with mild-to-moderate endometriosis. The main finding is that although there was an increase in pelvic pain scores from baseline across all three groups, the increase in pain magnitude (telehealth: +10 ± 12 mm, VR: +9 ± 24 mm) was not as large as that in the control group (control: +16 ± 12 mm). This result suggests that both digital health interventions may have the capacity to elicit a hypoalgesic effect on pelvic pain experienced by women diagnosed with endometriosis.

Our findings are consistent with a study which demonstrated that a 10–20 min VR session was able to alleviate pain in participants with chronic global pain [22] and endometriosis [15]. Jin et al. [22] showed that compared to the control group, the VR group had a 36.7% reduction in global pain scores during the intervention. Of note is the duration of the pain relief experienced, which only lasted for the duration of the VR session. However, similar to the current study, which indicated that pain reduction effects may persist up to 48 h post a single VR session, Merlot et al. [15] also found VR reduced pain perception beyond the treatment session (up to four hours post-VR session) in women with endometriosis. In addition, consistent with the present study, previous studies have also shown that telehealth-delivered exercise can alleviate knee pain in individuals with osteoarthritis [23], as well as chronic global pain in breast cancer survivors [24].

A plausible mechanism to explain the pain-relieving effect of VR- and telehealth-delivered exercise interventions may be their capacity to alter how pain-associated neurotransmission is processed in the central nervous system (CNS). The gate control and neuromatrix theories suggest that pain can be influenced by attention, emotion, and prior experiences related to pain [25,26]. It has been later hypothesized that pain perception requires attentional resources of the brain, which are finite [27]. Consequently, performing a task that consumes a lot of attentional resource, such as exercise for example, reduces the capacity for the processing of pain. A later study built upon this hypothesis by demonstrating that the regions of the brain involved in pain processing (i.e., insula, thalamus, hippocampus, midcingulate) decreased functional magnetic resonance imaging (fMRI) signal responses when participants directed their attention to a cognitively demanding Stroop task when administrated a noxious thermal stimulus [28]. These findings corresponded to the reduced pain intensities reported by the participants in that study.

The results of our study could also be explained by the interactions between the cardiovascular regulatory system and the pain system [29]. It has been shown in multiple studies that the activation of the baroreceptor reflex via an increase in blood pressure induces hypoalgesia [30]. It is hypothesized that the activation of the baroreceptor reflex not only triggers the pathway needed to reduce blood pressure as part of homeostatic regulation, but it also triggers inhibitory descending pain pathways. As such, this could potentially explain the decrease in pain experienced by the participants of this study after performing the exercise interventions (VR and telehealth).

However, it should be noted that there is one aspect of the VR study by Jin et al. [22] that is in contrast to the findings of the current study. While the current study did show a relative reduction in pelvic pain scores when comparing the intervention groups to the control group, there was still an absolute increase in pelvic pain scores in the intervention groups. This finding is in contrast with the study by Jin and colleagues [22], which showed an absolute reduction in the VR intervention group. The difference in these findings might be due to the different modalities of applications used in the VR intervention groups in both studies. The current study employed VR applications which contain exercise elements, while the study by Jin et al. [22] used Cryoslide, a non-exercise gaming VR application. According to a review by Koltyn [31], exercise produces a diminished sensitivity to noxious stimulation (hypoalgesia) following exercise, rather than inducing a complete absence of pain (analgesia). This is achieved through an increase in both pain thresholds and pain tolerances following exercise. As such, the relative reduction in pelvic pain scores but not an absolute reduction seen in the current study is consistent with the findings of this review.

### 4.1. Practical Considerations

Pelvic pain experienced by women with endometriosis has been shown to cause a negative impact on their functional and social well-being [32]. The strength of digital health tools resides in its ability to be delivered remotely, thereby increasing accessibility to this population. In addition, there is mounting evidence pointing towards the socio-economic benefits of digital health tools in managing various health applications [33]. Furthermore, the current study showed that both self-directed and supervised sessions were equally efficacious in relieving pain, thereby providing more options for clinicians in tailoring treatment options based on different individual needs and preferences. Together with the data presented in this study, there is an impetus for clinicians to consider the use of allied health professionals in the management of endometriosis. Specifically, a referral to an accredited exercise physiologist that could conduct exercise sessions via the utilization of digital health tools could potentially be of major benefit for women with endometriosis.

### 4.2. Study Limitations

There are several limitations to our study. Firstly, the sample size was not sufficient for the study to detect a statistical significance as the required number of participants needed to generate statistically significant results with an alpha of 0.05 was 30. This limitation exists as it was largely dependent on (1) the availability of respondents at the time and (2) restrictions imposed by the COVID-19 pandemic. Therefore, while the current study did manage to show an analgesic effect, it was incapable of demonstrating this via an absolute reduction in pain scores for both participants in both interventions, but rather via a relative reduction in pain scores compared to the control group. Further studies with a larger sample size will need to be conducted to confirm these findings.

Secondly, the VAS scale that was used in this study was modified from a previously validated VAS scale via the addition of numbered markings. To our knowledge, no other studies have used this version of the VAS scale. As such, it lacks reproducibility which may pose a possible limitation in our study. However, we believe that the numbered markings allowed the participants to produce a more objective assessment of their pain scores as it allowed them to compare their current pain scores to previous pain scores.

## 5. Conclusions

The main finding of this study was that a single bout of ‘self-managed’ VR-delivered exercise may be as efficacious as a ‘supervised’ telehealth-delivered exercise in reducing pelvic pain in women with mild-to-moderate endometriosis. Specifically, both digital health interventions were able to elicit a reduction in pain scores compared to the control group with a medium-to-large effect size. However, it should be noted that there was no statistically significant difference in pain scores between all three groups. Nevertheless, this pilot study provides important information on the efficacy of these digital health interventions and recruitment potential in this population group, and thus the feasibility of a larger and more expensive full-scale study.

## Figures and Tables

**Table 1:** Participant’s characteristics.

Variables	Telehealth (n = 7)	VR (n = 8)	Control (n = 4)
Age (years)	29 ± 7	27 ± 7	25 ± 4
BMI (kg/m^2^)	25 ± 4	23 ± 4	26 ± 2
Height (cm)	168 ± 10	169 ± 5	167 ± 6
Weight (kg)	70 ± 12	66 ± 12	72 ± 6
Time since laparoscopy			
More than 12 months (no. of participants)	3	4	3
Medications			
NSAIDS, (no. of participants)	2	2	2
Progestins (no. of participants)	4	5	4
Androgens (no. of participants)	1	1	0

## Data Availability

The data presented in this study are available on request from the corresponding author. The data are not publicly available due to ethical restrictions.
